# Neurodevelopmental heterogeneity and computational approaches for understanding autism

**DOI:** 10.1038/s41398-019-0390-0

**Published:** 2019-02-04

**Authors:** Suma Jacob, Jason J. Wolff, Michael S. Steinbach, Colleen B. Doyle, Vipan Kumar, Jed T. Elison

**Affiliations:** 1 Department of Psychiatry, University of Minnesota, Minneapolis, MN 55414 USA; 2 Department of Educational Psychology, University of Minnesota, Minneapolis, MN 55455 USA; 3 Department of Computer Science and Engineering, University of Minnesota, Minneapolis, MN 55416 USA; 4 Institute of Child Development, University of Minnesota, Minneapolis, MN 55455 USA

## Abstract

In recent years, the emerging field of computational psychiatry has impelled the use of machine learning models as a means to further understand the pathogenesis of multiple clinical disorders. In this paper, we discuss how autism spectrum disorder (ASD) was and continues to be diagnosed in the context of its complex neurodevelopmental heterogeneity. We review machine learning approaches to streamline ASD’s diagnostic methods, to discern similarities and differences from comorbid diagnoses, and to follow developmentally variable outcomes. Both supervised machine learning models for classification outcome and unsupervised approaches to identify new dimensions and subgroups are discussed. We provide an illustrative example of how computational analytic methods and a longitudinal design can improve our inferential ability to detect early dysfunctional behaviors that may or may not reach threshold levels for formal diagnoses. Specifically, an unsupervised machine learning approach of anomaly detection is used to illustrate how community samples may be utilized to investigate early autism risk, multidimensional features, and outcome variables. Because ASD symptoms and challenges are not static within individuals across development, computational approaches present a promising method to elucidate subgroups of etiological contributions to phenotype, alternative developmental courses, interactions with biomedical comorbidities, and to predict potential responses to therapeutic interventions.

## Introduction and autism’s history

In the 1940s, Kanner and Asperger separately published descriptions of patients who were aloof or withdrew from others and had socioemotional limitations in functioning^1,2^. Kanner highlighted that patients with autism insisted that things remained the same and were acutely upset when routines were changed. Although distinctions between Kanner’s early infantile autism and Asperger’s syndrome were made because of increased language, cognitive performance, and detailed knowledge in the Asperger’s group, each patient cohort highlights differences in intellectual abilities and their motivation for behavioral outcomes. In the early 20th century, dominantly held nurture-based theories blamed early maternal-child interactions and marginalized parents of affected children. Parent groups started to organize and advocate for children with autism by the 1960s^3^. Subsequently, autism research moved towards seeking biological etiologies and creating educational intervention strategies to increase functional capacities. Over the last forty to fifty years, the search for biological causes has largely focused on studying core elements of autism spectrum disorder (ASD). However, attempting to define a homogenous disorder group has been challenging.

ASD’s history captures many of the tensions between categorical and dimensional frameworks for psychiatric diagnoses. Traditional approaches rely on criteria lists that require clinicians to make dichotomous and categorical decisions, even though some individuals demonstrate significant symptoms that do not reach threshold for the disorder. Dimensional classifications and assessments conceptualize disorders as quantitatively rather than qualitatively different from a healthy state or a normative life course. There has been a historical sequence in autism classification, based on lumping or splitting its features based on clinical presentation, functional attributes, and genetic syndromes (e.g., Rett’s syndrome included in the Diagnostic and Statistical Manual of Mental Disorders, DSM IV)^4^. As early as the 1970s, genetic twin studies suggested strong heritability of the constellation of ASD symptoms^5,6^. However, specific genes were difficult to discover and clinical descriptions of “infantile autism” overlapped with childhood schizophrenia and psychosis in early versions of the DSM. More specific definitions of “autism” by the 1980s included impaired social communication and language, fear of change, and symptoms of odd interests manifesting before thirty months of age. Most ASD research to date has been designed with the categorical approaches defined by the DSM^4,7^ or the International Classification of Diseases^8^.

After the federal government made autism a special education category in 1990, they began collecting school data on identified children and quantifying access to special services. Children with high functioning ASD and Asperger’s often participated in typical classroom settings. Differences in defining Asperger’s syndrome continued even after it was added to the DSM in 1994^9^. Identification based on DSM definitions became more rigorous, valid, and reliable as diagnosticians captured detailed early developmental history from caregivers^10,11^ and tested social engagement through direct observational tasks such as the Autism Diagnosis Observation Schedule (ADOS) developed in the 1990s. These assessments became available commercially and have been increasingly used since the early 2000s^11,12^. Data collected has resulted in reclassification of symptom features and ASD’s behavioral subdomains. For example, the three domains of language communication, social deficits, and restrictive/repetitive behaviors were subsumed into two functional sub-areas of social communication and restrictive/repetitive behaviors^13,14^. Whereas the development of standardized tools has made categorical distinctions more valid and reliable, etiological discovery and treatment advances have continued to lag.

In this paper, we will discuss how ASD is currently diagnosed and what contributes to its clinical and neurobiological heterogeneity. We will also review computational methods that have attempted to streamline ASD’s diagnostic methods, distinguish differences from comorbidities, and follow developmentally variable outcomes. For example, supervised machine learning models train each data input with a corresponding target or known classification outcome, such as an existing diagnosis for ASD. Supervised methods may link data back to our already-existing categorizations. In contrast, unsupervised methods focus on many data inputs in order to find the structural relationships that occur between different inputs (e.g., symptoms/features that cluster together). Unsupervised methods allow us to identify new dimensions and categories from methods such as clustering approaches, factor analyses, and independent components analysis. This paper will highlight machine learning approaches from the marriage of computer science and statistics for pattern recognition applications in ASD. In addition, we will present an example of how ASD risk and symptom-related data can be ascertained within a community sample and analyzed using an unsupervised machine learning approach such as anomaly detection^15^. Specifically, it demonstrates how initial unsupervised methods could eventually use longitudinal feedback data for supervised methods to improve detection of early ASD signs. In sum, we will explore how novel computational methods with large datasets are particularly useful for studying complex neurodevelopmental disorders with multidimensional features and outcomes.

## Contributions to ASD heterogeneity

Determining how to effectively parse complex and overlapping features of a disorder with significant clinical variability has been an enduring challenge to the field of autism research. As a disorder, ASD exemplifies multidimensional processes because of its intra- and inter-subject clinical heterogeneity. Considered against other psychiatric disorders, ASD’s phenotypic variability is considerable^16,17^. Researchers are moving away from ASD as a unitary construct and viewing it as an umbrella term for multiple syndromes^18–20^, resulting from multiple varying etiological pathways^21^. There have been attempts to study subgroups of clinical subphenotypes (e.g., history of regression, presence of intellectual disability or limited language) in order to examine potential mechanisms^21–23^ and treatment targets. However, these approaches require increasingly large sample sizes in conjunction with refined and nuanced methods of subphenotyping^24–26^.

In addition, co-morbid clinical features add to the complexity of ASD characterization and presentation over developmental periods when other clinical populations or larger control groups are compared. In child psychiatry, co-morbidity or convergently arising diagnoses are common. Youth rarely have one disorder consistent with adult-defined phenomenological categories. Over a third of individuals with ASD meet criteria for attention deficit hyperactivity disorder (ADHD)^27^, obsessive compulsive disorder (OCD)^28,29^, disruptive behavior disorders (that includes oppositional defiant disorder)^30,31^, anxiety and mood disorders^31,32^, intellectual disability^33^, or epilepsy^34,35^. Other commonly reported co-morbidities include specific language disorder, constipation, and other known genetic and medical disorders^36,37^. Diagnostic trends have switched from viewing cognitive, language, compulsive, attentional, behavioral, mood and anxiety symptoms as part of the disorder to being named independently when they are severe enough to warrant specific treatments^27,38^. Child psychiatrists have long known that certain disorders frequently emerge and present together. For example, ADHD, OCD, and Tourette’s or tic disorders^39^ as well as oppositional defiant disorder, ADHD, and minor depression/dysthymia^40^ are common triads. These often present by early school age and with varying severity of symptom clusters in boys versus girls. Some disorders may have earlier signs, but diagnoses are made when children struggle to reach or maintain expected milestones at school or home. Various diagnostic combinations occur with ASD and specific convergent diagnoses are increasingly being identified. Treatments often require modification based on cognitive features of ASD. Research studies that exclude other psychiatric disorders have limited application in the community because of the pathophysiological overlap between ASD and many comorbid disorders.

## Computational psychiatry and new approaches to studying ASD

Computational psychiatry is of growing interest because it uses mathematical approaches to quantitatively investigate interacting variables across biobehavioral system levels within and between psychiatric disorders. As a newly emerging conceptual approach, it covers a range of strategies to characterize and investigate complex and interacting phenomena that contribute to outputs such as clinical presentation of neurobehavioral disorders. Computational methods can be applied at multiple levels in psychiatry by improving behavioral and biological diagnostic approaches (e.g., diagnostic or treatment-related biomarkers) and to subcategorize brain and behavioral dysfunction through the use of large datasets. For example, methods may be used to model neural circuits by accounting for multifactorial contributions (e.g., genetic and environmental factors) as explicit mathematical terms in order to test hypotheses about how multiple variables affect circuit function.

Time and progression of a disorder are important because psychiatric disorders present differentially across the lifespan and are nonlinearly influenced by biological processes related to growth, reproduction, or degeneration. Computational models in psychiatry have the potential to test how circuit or biological dysfunction at an initial time interval could create progressive disruptions through alterations in neural development and plasticity. These approaches have the potential to characterize individual differences required to ascertain “what is different about” how this specific child at this time “processes information about the world”; this is required to tailor biobehavioral interventions for subgroups^41^. As our technological ability to capture and share data increases, neural and other biological variables collected over time may be used to sequentially predict and discern behavioral outcomes at the level of the individual. Ultimately, computational and machine learning approaches will help subgroup multifactorial inputs and outputs in order to create specific treatment plans for individual children with ASD and other developmental disorders.

### Machine learning approaches used to identify key diagnostic features of ASD

Highly standardized ASD assessments require more evaluation time than most psychiatric disorders and a high level of clinical training with ongoing reliability confirmation. As the need for assessments increase, care providers seek to decrease redundant measures and minimize the time to complete separate instruments. Given the range of signs and symptoms listed in the DSM, questions arise about whether some features are more important and central to the diagnostic category. Researchers are now utilizing large datasets required for genetic studies and analyses to address such concerns.

Several studies have evaluated machine learning as a means to shorten the clinician-expert administrated ADOS assessment, to test the accuracy of an observation-based classifier for rapid detection of autism risk, or to detect a minimal set of behaviors through feature selection-based algorithms^42–44^. An early machine learning classifier of scored behaviors reported 99.7% sensitivity and 94% specificity using 8 of the 29 items contained in ADOS Module 1^44^. Although limiting items reduces testing time, this approach fails to consider that these expert testers were already drawing from broader information and their high level of training in diagnosis. For example, clinical evaluators integrate multifaceted information from their full encounters and do not assess subtest sections in isolation. Later, the authors retested the 8 items in subsequently larger datasets (autism = 1884, broader ASD = 449, and 283 non-ASD diagnoses) and reported sensitivity of 97.1% and specificity of 83.3%^44^. They attributed the lower specificity to the small number of controls used in the earlier study. The 8 items do not robustly produce optimal performance across each dataset previously combined to create the large sample^45^, suggesting that information from some of the remaining 21 items were also valuable. Subsequently, this group examined modules 2 and 3 of the ADOS, which are appropriate for individuals with higher language and cognitive abilities^43^. They reported between 98.3 and 97.7% accuracy using 9 of the 28 items from module 2 and 12 of the 28 items from module 3 to be sufficient to detect ASD risk, respectively.

Across all three ADOS focused studies described above, atypical eye contact, facial expressions (e.g., social smile in Module 1), interaction enjoyment, and joint attention were key features of ASD. In the modules requiring higher language and cognitive functioning, use of gestures, social communication or conversation, quality of social overtures, amount of reciprocal interactions, atypical motor mannerisms, and restricted/repetitive interests were also important features^43^. These studies suggest that cognitive level and daily functional abilities influence how many and what symptoms inform a diagnosis. This work also highlights that developmental level differentially influences the contribution of individual items. Future studies will be needed to account for chronological or adaptive age in streamlined diagnostic algorithms.

Data from detailed early developmental parent interviews obtained from the revised Autism Diagnostic Interview (ADI-R) were also investigated using machine learning methods. Wall and colleagues^46^ tested the accuracy of a 7-question classifier (reduced from 93 items of clinician-expert interview scores) in research datasets with the full standardized parent interview. Bone et al.^45^ were not able generate comparable findings when they used a larger dataset with more controls and severely affected ASD participants. In a follow up study, Bone and colleagues^47^ used machine learning to generate screening questions using the ADI-R and the Social Responsiveness Scale (SRS)^48^. Both sensitivity and specificity were differentially weighted to achieve near-peak performance with five or fewer codes using Machine Learning-based fusion of ADI-R and SRS items. A screener algorithm for under versus over 10 years of age reached 89.2% (>10 years, 86.7%) sensitivity and 59.0% (>10 years, 53.4%) specificity for five behavioral codes. Note that demarcating age is important here and that items vary in importance over the developmental time course. The most frequently coded ADI-R items that overlap across papers include reciprocal conversation, direct gaze, and group play with peers. Authors highlight that it is possible to create robust, customizable screening or diagnostic instrument algorithms^47^. However, outcomes are different when controls with other difficulties or co-morbidities are included and age cutoffs are varied. Future testing with screening items alone in a community-based population versus a research clinic sample will be required to confirm the effectiveness of prioritizing specific or temporal features of ASD.

In addition to this diagnostic testing literature, there are prospective studies on neuroimaging of infants at high familial risk for ASD. For example, Emerson and colleagues^49^ utilized resting-state functional magnetic resonance imaging (MRI) and a cross-validated machine learning algorithm applied to the imaging data collected at age 6 months to predict diagnostic outcomes at age 2 years. They reported a positive predictive value of 100% and negative predictive value of 96% and functional connections with social communication and repetitive behavior at age 2 years. See reviews of the literature on imaging and early identification of ASD^50^, as well as limitations of use of machine learning approaches with limited sample sizes in many current neuroimaging studies^51^.

### Machine learning approaches may be used to compare frequent comorbidities and convergencies, such as ASD and ADHD

It is estimated that between 30–80% of individuals with ASD meet ADHD criteria^52^. The diagnostic time frame may overlap but tends to be later for identifying ADHD, which is more often noted with increased attentional demands required for abstract and analytical thinking in elementary education. DSM-5 modified symptoms being detected for ADHD prior to twelve years of age versus the earlier seven years that was required for DSM-IV-TR. ADHD also has subdomains of inattention and hyperactivity/impulsivity. Some research has attempted to clarify overlapping and unique patterns of cognitive impairment for children with ASD versus ADHD^53^.

Research on ADHD alone has attempted to integrate behavioral and/or phenotypic information with brain functional and structural MRI. Anderson and colleagues^54^ used four Non-Negative Matrix Factorization algorithms to find the best fit for subnetworks that clustered with the ADHD-Inattentive diagnosis. Brain areas highlighted were the posterior cingulate, precuneus, and parahippocampal regions. Authors concluded that multimodal data in ADHD (*N* = 730) can be interpreted by latent dimensions and unsupervised computational approaches, adding to a growing number of studies using supervised computational approaches^54^.

Few studies have attempted cross-diagnostic classification across ASD and ADHD. One study, conducted by Lim and colleagues, reported high accuracy when discriminating ADHD from controls versus ASD (accuracy 85.2 vs. 79.3%) when applying a Gaussian process classification to gray matter volumetric data^55^. Another study considered both ASD and ADHD, but only compare each classification against controls and not with each other^56^. They used automated classification based on histograms of oriented gradients features extracted from MR brain images. Authors reported hold-out diagnostic accuracy ranged from 65.0–69.6% (over baseline 51.6–55.0%) in ASD and ADHD, respectively.

Using behavioral rating data, another study^57^ attempted to distinguish between ASD and ADHD by using different machine learning classification scores from the 65-item SRS. They tested six machine learning models on ASD (*N* = 2775) or ADHD (*N* = 150) individuals, reporting that five of the 65 behaviors measured were sufficient to distinguish ASD from ADHD (area under the curve = 0.965). Challenges with these studies occur because of the difficulty in subcategorizing the >20% number of children with ASD who also have significant ADHD.

In a recent review by Uddin et al.^58^, they summarize machine learning neuroimaging approaches from both populations. For ASD they reviewed 29 neuroimaging-based classification studies, and report how functional connectivity, gray matter volume, and default mode network approaches are being used to discriminate ASD from typical development. For ADHD alone, they reviewed nineteen studies showing that areas are more widespread but frontal and cerebellar regions appear to be important for classification compared to typical development. Obstacles for reliability and reproducibility include challenges of clinical heterogeneity in populations and standardization of data acquisition methods across sites. Addressing such heterogeneity is consistent with new research initiatives that are motivated to find biologically homogenous profiles of impairment. Identifying the structural and functional network signatures of multi-dimensionally-defined developmental profiles using computational psychiatry has the potential to move us toward a more biologically informed nosology, consistent with current research initiatives^41,59–61^.

### Computational approaches used to study longitudinal changes in ASD

With increasing follow-up and standardized longitudinal data on individuals with ASD, computational methods are helpful for predicting outcomes by characterizing developmental trajectories. In computational models of neurodevelopmental disorders, time is an important variable because it captures sensitive and critical periods of growth that influence functional outcomes.

Lord and colleagues^62^ have published a series of papers examining trajectories of change in symptoms over the developmental course of ASD. In a clinic-referred population, latent class growth curve models assessed longitudinal data from 2 to 15 year olds (*N* = 345). The best-fit model identified a large subgroup (80%) with the stable high or stable moderate severity of ASD symptoms and two smaller groups with increases (9%) or decreases (7%) in severity over time. Although age, gender, race, and nonverbal IQ did not predict group membership, verbal IQ was maintained or increased over time in all groups and adaptive behavior worsened in all groups (except the small improving group). More recently, Lord and colleagues completed a growth curve analyses with a very detailed longitudinal follow-up (*N* = 85) of developmental trajectories from age 2 to 19 grouped by outcome^62^. Although the sample size was small, groupings were based on 19-year-old outcomes of verbal IQ, nonverbal IQ, social adaptive skills, and parent-reported social-communication. Differences in childhood trajectories for more or less cognitively able children were plotted over time beginning at age 2. Linear (Nonverbal IQ, ADI-R Repetitive Sensory Motor and Insistence on Sameness) and quadratic (Verbal-IQ, Vineland Social Adaptation, ADI-R Social Deficits) growth curves were shown. Differences in independent functioning and lack of comorbidity were associated with preschool through adolescent trajectories in social adaptation, social deficits, and insistence on sameness. Of note, change in social adaptation and decreased insistence on sameness distinguished ASD with higher cognitive abilities by adulthood from those with lower IQ outcomes. A small group of young adults who had childhood diagnoses of ASD (*N* = 8) with IQs in the average range were functioning socially and adaptively at age-appropriate levels.

Another study followed a cohort of children with ASD (*N* = 152) at three discrete time points and a subset of outcome measures over a ten year period^63^. Two distinct but parallel trajectories were identified for adaptive behavior and daily living skills. For social and communication, one trajectory showed increased growth while a flatter trajectory for adolescent outcomes was observed when participants started with lower cognitive and language skills, early epilepsy, and more severe ASD symptoms around age five.

More recently, another study reported longitudinal data (*N* = 105 children with ASD) using growth mixture model analysis with four assessments between the ages of 3–8 years^64^. Best-fit models produced one decreasing trajectory (73% of sample) and another moderate and stable class (27%) using a standardized adaptive functioning measure (Vineland-II)^65^. Focusing only within the preschool years, a multisite Canadian study used a semiparametric group-based approach to identify distinct mixtures of trajectories of ASD children (*N* = 421) over four time points (baseline, at 6 months and 12 months after baseline, and at age 6 years)^66^. Best-fit models showed an improvement in adaptive functioning in approximately 20% of the sample. In contrast, ASD symptom severity was more stable and only 11% of the sample showed a decrease in symptom severity. Taken together, these findings confirm that we have limited data over extended developmental periods, that outcome measures are inconsistent across studies, and that sample sizes need to be larger to better characterize heterogeneous trajectories of development with ASD.

### Machine learning approaches to study ASD utilizing large or biologically defined datasets

In contrast to the highly specialized and intensive resources required to follow a clinical sample over time, another computational approach is to use large existing datasets that store health information as it was accessed. For example, electronic medical record time series analyses (6 month windows from birth to 15 years old) were used to examine comorbidities with ASD^67^. Hierarchical clustering methods were used to identify four groups (defined by salient features that included seizure, psychiatric, auditory, gastrointestinal) that were distinct from the larger sample and that could not be attributed to another medical cause. Three patterns of medical trajectories were identified using an unsupervised approach. Only the gastrointestinal and seizures disorder groups had between group correlations with both symptoms, and this finding was replicated in another sample population. Future research may use these methods of subgrouping to examine etiological risk factors related to ASD, including genetically-linked subgrouping based on specific comorbidities^68^.

Scientists often seek “causal” determinants that make disorders easier to classify in a binary categorical manner and to find targeted treatments or cures. For example, the gene required for Rett syndrome was identified when comparisons were made to the broader category of “idiopathic autism”^69,70^. Other studies have examined ASD phenotypic presentation and overlap in fragile X and Prader-Willi syndrome (PWS). A recent paper^71^ reported that 51% of males versus 18% of females with fragile X syndrome (FXS) have co-morbid ASD as assessed by the Social Communication Questionnaire (SCQ). This comorbid subgroup had a higher prevalence of seizures, more sleep and behavior problems, and similar side effect profiles with some medications. They were underserved with behavioral treatments offered to children with “idiopathic” ASD. In contrast, 12.3% of children with PWS had ASD according to the ADOS-2 versus the 29–49% that screened positive for ASD with the SCQ^72^. Communication problems were observed in positive screens that did not make clinical diagnostic cut-off criteria. Genetic specificity was observed because the majority with ADOS-2 confirmed ASD also had maternal uniparental disomy PWS genetic subtype. This approach requires very large datasets in order to chip away at discovering small subgroups of the broader phenotype that can be attributed to specific genetic causes.

Although refined subgroup characterization promises to identify genetic or other etiological subtypes, the wide net of broader autism phenotype captures features of the many neurodevelopmental genetic syndromes altogether. Studies focusing on subgroups may be useful in understanding brain development across a broader population. For example, large clinical samples required to do genetic studies of ASD have recently been used to also study spatiotemporal development in the brain^73^. Authors calculated expression signatures specific to spatiotemporal windows (16 brain regions and 13 developmental stages). In order to identify when and where predicted ASD genes are specifically active, their analytic approach required carefully controlled permutation tests. There are large gaps of knowledge between causal and contributing genes and common neural networks that need to be targeted for educational and clinical interventions across overlapping phenotypic clusters.

Advances in precision or personalized medicine will require an ability to disentangle the complexity of overlapping symptoms in order to identify neurobehavioral pathways that specifically impact functional outcomes. For example, ASD researchers have struggled to identify circuit pathways or molecular mechanisms that lead to specific treatment targets because subgroups are influenced by additional dimensions of variability (e.g., attention deficits, intellectual disability, or severity of insistence on sameness). Moreover, sampling approaches intending to create “clean” study samples by excluding participants with other disorders have produced mixed outcomes. Clinicians already know that research methods optimized to increase homogeneity and reduce heterogeneity have limited utility for translating research outcomes to real-world communities. Long-term approaches will benefit from increased sample sizes of typical and atypical data from children to model developmental trajectories of ASD. This would increase our ability to find the weak links or pathways that lead to systemic and specific functional impairments^41^ as children face incremental challenges with age.

Given that ASD is known to emerge during the first years of life, understanding variability in early typical versus atypical development is likely to yield particularly important insights regarding heterogeneity. The data presented below uses a community sample that can be followed over time and a novel computational approach to examine risk for developing ASD-related symptoms. Focusing on early development is an essential step for creating and selecting treatments that target plastic neural systems and compensatory processes unique to this period.

## Proof of principle: anomaly detection as a computational example for detecting ASD risk and variance in a community sample to be followed longitudinally

Anomaly detection focuses on identifying data that markedly deviates from the normal patterns that are observed within datasets. Although statistical approaches have detected outliers or anomalies since the 19th century^74^, current methods have advanced by integrating machine learning, data mining, information theory, and spectral theory in order to tackle specific data questions^15^. Atypical observations sometimes group together in clusters, but those clusters are often relatively small and less cohesive, and thus specialized techniques for finding anomalies have been developed in a wide variety of disciplines^15,75^. Multiple anomaly detection techniques are available, including techniques such as local outlier factor (LOF)^76^, one class support vector machines^77^, and autoencoder neural networks^78^.

Here, we demonstrate how unsupervised anomaly detection is used to identify early risk features that may predict autism or related disorders in early development. The data is from a community sample of data gathered from 1570 children between 17–25 months of age. Methods used have been previously described^79,80^ for online acquisition^81^ of the Video-Referenced Rating of Reciprocal Social Behavior^82^, the Repetitive Behavior Scale for Early Childhood^80^, and the MacArthur-Bates Communicative Development Inventories^83^, along with demographic information. Over time, subsamples of these toddlers will be recruited to complete an independent follow-up evaluations with researchers blind to the online assessment data from the first time point. This longitudinal data will be used to give feedback information and to improve working models of early developmental heterogeneity related to autism versus other neurodevelopmental outcomes.

**Fig. 1:**
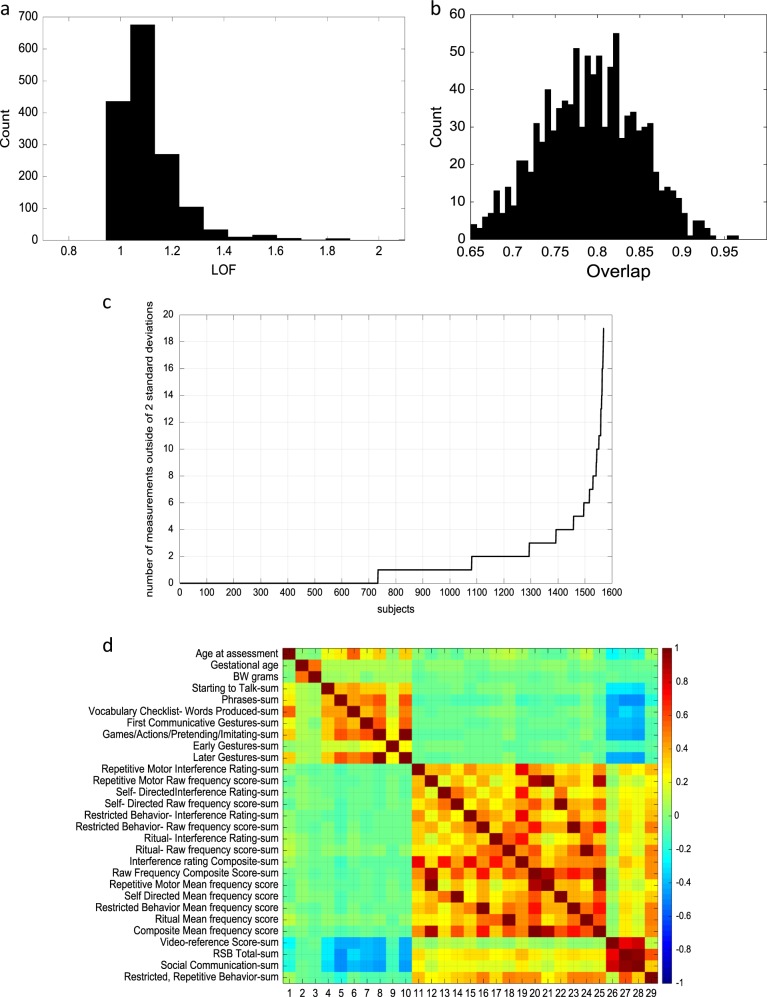
**a** Distribution of local outlier function (LOF) scores. Scores greater than 1.32 are classified as anomalies. Although histograms are 1-dimensional, scores are calculated from the data records of each participant which are multidimensional. As an analogy, the distance between two points in 3- dimensional space is a 1-dimensional number. As a test, if the 80 anomalous toddlers are omitted and LOF scores are recomputed, only 6 subjects obtain LOF scores greater than 1.32. Note these 6 had initial LOF scores near the 1.32 threshold. For this example, 5% was chosen as the LOF threshold because it corresponded to a point at which the LOF distribution changed from a “bump,” i.e., a somewhat normal looking distribution to a more uniform distribution. Such a region represents a different and lower density area of the data space where anomalies are expected to be found. For other datasets, the appropriate LOF threshold might correspond to a different percentage of the data, e.g., 1 or 10%. As an example of an unsupervised anomaly detection approach, we were able to determine that 80 children had LOF > 1.32 scores out of the overall sample of 1570 toddlers. **b** Overlap between anomalies detected in full sample vs. 1000 random samples of half the data. Many anomaly detection routines are also relatively robust to sample size. To illustrate this, we randomly sampled the data set to obtain 1000 half size samples. The overlap in anomalies found in the samples with the full data set is shown. On average, 74% of the anomalies (LOF > 1.32) found in the half size samples occurred in the full data set. Furthermore, the correspondence between the LOF scores in the full sample and the half sized sample is about 91%. On average 94% of the anomalies (LOF > 1.32) found in the half size samples have an LOF score of 1.22 or more in the full data set. All of this is a sign that LOF scores are relatively stable for the sample under consideration. The goal of this evaluation was to test how much the LOF score depended on the sample size and thus, whether a threshold determined as anomalous in one sample could be used for a smaller sample. More generally, we evaluated how much the LOF score varied by comparing the LOF scores of a subject in the half size samples to the LOF score in the full sample. **c** An alternative way to view the sample data from LOF scores shows number of subjects outside of two standard deviations. **d** In addition, the data can be plotted to illustrate the levels of correlations between variables assessed in a matrix plot. Multiple waves of data collection for ages 18–24 months, would allow for testing and retesting of the reliability of these patterns with these dimensions of observations as the sample size increases

In this example, anomaly detection is initially being used to calculate how each parent rating about their child deviates across normal dimensional patterns of behavior. This approach produced anomaly scores that fell into a relatively small range around a central peak, with “true” anomalies extending into a rightward tail. Routines were considered robust after a process of checking the data. When anomalous cases were eliminated from the full dataset of 1570 children, few to no new anomalies were detected when the routines were rerun. To illustrate this process, we computed LOF anomaly scores^76^. Note that the scores incorporate information across many items and dimensions of behaviors. As represented in Fig. 1a, we identified 80 out of 1570 toddlers (or 5%) with an LOF score greater than 1.32, which we chose (for this example) as the threshold for being anomalous (95th percentile of LOF scores).

### How accurate or stable is this computational model in identifying anomalies with these behavioral variables?

Figure 1 shows a histogram of LOF scores. Although it looks 2-dimensional in this plot, the LOF scores are calculated from many variables of each participant, which indeed represents multiple dimensions of behavior. For example, the distance between two points in 3-dimensional space is a 1- dimensional number. The accuracy of LOF threshold score can thus be tested. If 80 anomalous toddlers are omitted and LOF scores are recomputed, only 6 subjects obtain LOF scores greater than 1.32. Note these 6 had initial LOF scores near the 1.32 threshold. This suggests that this threshold is fairly accurate when all these behavioral variables are considered together (see Fig. 1d for listing of variables). For this dataset, 5% was chosen as the LOF threshold because it corresponded to a point at which the LOF distribution changed from a “bump,” i.e., a somewhat normal looking distribution to a more uniform distribution. Such a region represents a different and lower density area of the data space where anomalies are expected to be found. For other datasets, the appropriate LOF threshold might correspond to a different percentage of the data, e.g., 1 or 10%. Using this approach, we are able to test if an appropriate LOF threshold is identified for a particular population based on how the distribution is affected when anomalies are removed.

### How accurate or stable is this computation with this sample size?

Many anomaly detection routines are also relatively robust to sample size. To test this in our data, we randomly sampled the data set to obtain 1000 half size samples. The overlap in anomalies found in the samples with the full data set is shown in the Fig. 1b. On average, 74% of the anomalies (LOF > 1.32) found in the half size samples occurred in the full data set. Furthermore, the correlation between the LOF scores in the full sample and the half sized sample is about 91%. On average 94% of the anomalies (LOF > 1.32) found in the half size samples have an LOF score of 1.22 or more in the full data set. These are signs that LOF scores are relatively stable for this current sample under consideration. Moreover, it suggests that the threshold determined as anomalous in a large sample could also be used for a smaller sample. Given our focus on heterogeneity, we are also able to evaluate how much the LOF score varied by comparing the LOF scores of a subject in the half size samples to the LOF score in the full sample.

### How does this sample relate to previously collected population data?

With the initial developmental data, we were able to determine that 80 children had LOF > 1.32 scores out of the overall sample of 1570 toddlers. This represents about 5% of the sample. This percent of outliers is between the prevalence data of 13% for developmental disabilities and the 1.5–2% estimates for prevalence data of ASD^84,85^. Longitudinal follow up will confirm if around 3% of this sample will have closely related developmental disorders affecting language or developmental delays that do not fulfill full criteria for autism. There are different ways to view and select outliers and anomalies from typical development. An alternative way to view this data is to look at the number and identity of individuals outside of two standard deviations across variables (see Fig. 1c). Another way to view relationships between variables is to plot correlations between the variables assessed (Fig. 1d).

### What are way to improve predictive models with a longitudinal study design?

Next steps would be test how predictive and accurate this unsupervised approach to identify children at risk is for later diagnoses of autism or related developmental disorders (e.g., developmental delay, specific language disorder, etc.). As we follow these children longitudinally, we will use supervised computational models to augment what is initially learned from the initial anomaly detection analyses. Expert examination and tracking outcome diagnosis back to the anomaly detection results will help refine the model. Multiple waves of data collection for ages 18–24 months, would allow for testing and retesting of the reliability of this computational approach as the sample size increases. Different trajectories may be observed. Subgroupings of anomalous subjects with similar clinical features may be identified earlier and be linked to similar etiologic factors or genetic risk.

Supervised anomaly detection approaches use class labels, but have the same goal of distinguishing anomalous and normal points. More generally, some supervised anomaly detection approaches produce an anomaly score that, as with unsupervised anomaly detection, allow for the ranking of data points according to how anomalous each clinical feature is. Combined with an understanding of the anomaly detection algorithms, supervised feedback could be used to refine the algorithms and filter the results for those relevant to identifying subjects at high risk for ASD. Recent implementations of anomaly detection approaches have been able to detect patterns and outliers in sequence data^86,87^.

These advances will afford the opportunity to model complex, sequential data such as those observed in neurodevelopmental disorders. Other analytical methods can be tested and compared with the results of anomaly detection methods, including clustering techniques such as k-means, hierarchical, and shared nearest neighbor^88^. Alternative supervised approaches such as ensemble techniques^89^ may be employed to further incorporate the clinical symptom observations as feedback. The utility of such approaches for early identification of autism risk needs to be confirmed through future research. Community samples that include typically developing children as well as children at risk for a range of neurodevelopmental disorders may be helpful to develop population-based methods for early detection of disorder risk.

## Next steps and conclusions

The complex, heterogeneous nature of ASD has impeded our efforts to understand etiology and to predict which treatments will be effective. Big data and machine learning approaches may not only serve to parse subgroups within a large heterogeneous clinical category, but may also be used to examine common treatment targets across distinct neurodevelopmental trajectories. In such computational studies, samples need to be large enough for training and retesting computational models as they are optimized. The goal is to capture as much of the variation of the disorder as possible and conduct analyses to delineate biologically and clinically meaningful subgroups. An advantage of computationally driven research is the ability to compare multiple analytic methods to hone our ability to predict outcomes and quantify risk in the midst of heterogeneity.

Although large data-driven approaches require multidisciplinary collaboration and investment, they are increasingly important given the complexity and heterogeneity associated with developmental disorders such as ASD. We have learned through over 70 years of research that ASD defies simple categorical classification. This necessitates new approaches that leverage larger samples to build reliable models that accurately reflect the complexity inherent to autism. This “complexity” refers not solely to inter-subject variability, but also to intra-subject phenotypic variability as a function of development. Because ASD symptoms and challenges are not static within individuals across development, computational methods may contribute to better understanding of growth and time-related courses, subgroups of etiological contributions to phenotype, and interactions with medical-psychiatric comorbidities.
